# Octanoic Acid and Decanoic Acid Inhibit Tunicamycin-Induced ER Stress in Rat Aortic Smooth Muscle Cells

**DOI:** 10.1155/adpp/9076988

**Published:** 2024-11-26

**Authors:** Wanida Sukketsiri, Varomyalin Tipmanee, Panlekha Rungruang, Mayo Higashihara, Tomoko Sumi, Tatsuya Moriyama, Nobuhiro Zaima

**Affiliations:** ^1^Division of Health and Applied Sciences, Faculty of Science, Prince of Songkla University, Songkhla, Thailand; ^2^Department of Biomedical Sciences, Faculty of Medicine, Prince of Songkla University, Songkhla, Thailand; ^3^Molecular Medicine Program, Multidisciplinary Unit, Faculty of Science, Mahidol University, Bangkok, Thailand; ^4^Department of Applied Biological Chemistry, Graduate School of Agriculture, Kindai University, Nara, Japan; ^5^Agricultural Technology and Innovation Research Institute, Kindai University, Nara, Japan

**Keywords:** antioxidant, CHOP, eIF2*α*, ER stress, GRP78, medium-chain fatty acids

## Abstract

ER stress is a crucial factor in the progression of vascular cell diseases. Notably, octanoic acid (OA; C8:0) and decanoic acid (DA; C10:0), prominent components of medium-chain fatty acids (MCFAs), may provide potential health benefits. However, their effects on vascular smooth muscle cells (VSMCs) remain unknown. Given the link between ER stress and vascular cell pathological conditions, the primary goal of this research is to investigate the protective effects of OA and DA against ER stress induction in rat aortic smooth muscle cells (RASMCs). To achieve this objective, RASMCs were pretreated with OA and DA at concentrations of 250 and 500 μM for 24 h. Subsequently, the cells were exposed to 1 μg/mL of tunicamycin, an ER stress inducer, for an additional 24 h. Apoptosis was assessed using DAPI staining, while DCFH_2_-DA probe was used to measure ROS levels. Furthermore, the gene expression of ER stress markers, such as *CHOP*, *GRP78*, *ATF4*, and *eIF2α*, as well as contractile markers like *αSMA* and *MYH11*, was assessed using real-time reverse transcription polymerase chain reaction. Moreover, the *α*SMA protein level was measured using immunocytochemistry techniques. The study revealed that OA and DA significantly mitigated cell death caused by tunicamycin, decreased ROS production, and inhibited the gene expression of ER stress markers (*CHOP*, *GRP78*, and *eIF2α*). Notably, OA and DA also inhibited the expression of contractile genes (*α-SMA* and *MYH11*) and reduced the number of *α*-SMA-positive cells in tunicamycin-treated RASMCs. These findings indicate that OA and DA offer protection against ER stress–stimulated cell death and ROS generation in VSMCs, thereby supporting their potential therapeutic applications for safeguarding these cells.

## 1. Introduction

Acute aortic dissection and ruptured aortic aneurysms are well-established leading causes of death in cardiovascular disease [1]. They are characterized by the progressive enlargement of the aorta, which can ultimately lead to acute dissection or rupture. These conditions are often associated with abnormal hemodynamic loads, primarily induced by hypertension, and the weakening of the vascular wall [2]. Several studies have noted a significant decline in the vascular smooth muscle cell (VSMC) number within the vascular wall of aortic dissections [3, 4]. Furthermore, α-SMA and MYH11 are crucial components of VSMCs that contribute to the structural integrity and function of the aortic wall. Mutations in these genes can lead to dysfunction in VSMCs, contributing to the pathogenesis of aortic aneurysms through mechanisms such as reduced contractility, altered extracellular matrix remodeling, and increased susceptibility to mechanical stress [5–7]. Understanding these molecular pathways is essential for developing targeted therapies for the management and prevention of aortic aneurysms.

ER stress occurs when there is an imbalance in the unfolded protein response and the regulation of ER homeostasis. This imbalance can have significant effects on various biological and pathological processes [8]. Alterations in ER function, accompanied by the accumulation of misfolded proteins, can trigger the onset of ER stress. Research has consistently shown that ER stress is linked to the development and progression of cardiovascular diseases, including aortic aneurysm [9]. In the walls of aortic aneurysms, an increase in ER stress markers, such as CHOP, GRP78, and eIF2A, has been observed. This increase promotes the apoptosis of VSMCs, endothelial dysfunction, and inflammatory infiltration and ultimately leads to the formation of aneurysms [10].

Octanoic acid (OA; C8:0) and decanoic acid (DA; C10:0) are a class of saturated fatty acids, primarily composed of medium-chain fatty acids (MCFAs) [11]. These fatty acids are primarily found in human milk fat and several plant oils, for example, coconut oil and palm kernel oil [12–14]. MCFAs are readily absorbed and provide a rapid energy source. Recent research suggests that MCFAs may have pharmaceutical and biological potential with low toxicity [15]. Numerous studies have demonstrated the beneficial effects of a high MCFA diet on lipid metabolism and accumulation in rats afflicted with nonalcoholic fatty liver disease. This regulation occurs through the modulation of apoptosis, oxidative stress, and inflammatory responses [16]. Moreover, an additional study conducted by Zhang et al. [17] found that MCFAs effectively mitigated cell injury induced by lipopolysaccharides by downregulating necroptotic and inflammatory signaling pathways. While the health advantages of MCFAs are well-established, there is limited information available regarding the impact of MCFAs on ER stress and its relevance to VSMCs. Thus, this study aims to investigate the protective effects of OA and DA against ER stress induction in RASMCs and to elucidate the underlying mechanisms.

## 2. Materials and Methods

### 2.1. Cell Culture

RASMCs and RAW 264.7 macrophage cell lines were maintained under high glucose Dulbecco's Modified Eagle Medium (DMEM) (Nacalai Tesque, Inc., Kyoto, Japan) supplemented with 10% (v/v) fetal bovine serum (Nacalai Tesque, Inc.) and 1% antibiotic (penicillin/streptomycin; Nacalai Tesque, Inc.). The cells were grown in a humidified tissue culture incubator at 37°C with a 5% CO2 atmosphere. Both cell types play a critical role in the pathology of abdominal aortic aneurysm.

### 2.2. Cytotoxicity Assay

RASMCs (10^4^ cells/well) and RAW 264.7 (10^4^ cells/well) cells were plated into 96-well plate under complete high glucose DMEM for overnight. Both cells were treated with OA (Nacalai Tesque, Inc.) and DA (Nacalai Tesque, Inc.) at the same concentration (1, 50, 100, 250, 500, and 1000 μM) for 24 and 48 h. The cytotoxicity of test compounds was assessed by using 3-(4,5-dimethylthiazol-2-yl)-2,5-diphenyltetrazolium bromide (MTT; Wako Pure Chemical Industries LTD., Osaka, Japan) assay and the absorbance of each well was detected with a microplate reader (Bio-Rad Laboratories, Inc., California, USA) at 570 nm.

### 2.3. Cytoprotective Effects of OA and DA Against Tunicamycin-Induced RASMCs by MTT Assay

RASMCs (10^4^ per well) were seeded into 96-well plates overnight, followed by pretreatment with OA and DA at 250 and 500 μM for 24 h. Following this, the cells were cultured in serum-free medium and exposed to tunicamycin (1 μg/mL) in the presence or absence of OA and DA at concentrations of 250 and 500 μM for an additional 24 h. Afterward, the cells were incubated with MTT solution for 4 h and then measured at 570 nm using a microplate reader.

### 2.4. Antiapoptosis Activity of OA and DA Against Tunicamycin-Induced RASMCs by DAPI Staining

RASMCs (5 × 10^4^ per well) were grown in 8-well slide chamber (Watson Bio Lab, Kyoto, Japan) overnight and then pretreated with OA and DA at 250 and 500 μM for 24 h. After 24 h of pretreatment, RASMCs were induced with tunicamycin (1 μg/mL) with or without OA and DA at 250 and 500 μM for 24 h under serum-free medium. Following the treatment, the cells were added with 4% paraformaldehyde (PFA) to fix the cells and then stained with DAPI (LGC Seracare, USA) for 30 min. Images were captured using a Nikon ECLIPSE E200 fluorescence microscope (Nikon, Tokyo, Japan). The percentage of apoptotic cells was determined by dividing the number of apoptotic cells by the total number of cells and then multiplying the result by 100.

### 2.5. ROS Production by 2,7-Dichlorodihydrofluorescein Diacetate (DCFH_2_-DA) Assay

RASMCs (5 × 10^4^ per well) were cultured into the 8-well slide chamber overnight and then pretreated with OA and DA at 250 and 500 μM for 24 h. Subsequently, they were induced with tunicamycin (1 μg/mL) in the presence or absence of OA and DA for another 24 h under serum-free medium. After the treatment, the cells were fixed with 4% PFA and then stained for intracellular ROS production using DCFH_2_-DA. The cells were incubated with DCFH_2_-DA (50 μM) for one hour in dark conditions. The fluorescence of 2,7-dichlorofluorescein (DCF), which is the end product of DCFH_2_-DA, was observed with a fluorescence microscope. The intensity of DCF was subsequently analyzed using the ImageJ software (https://imagej.nih.gov/ij/download.html).

### 2.6. Immunocytochemistry Assay

*α*-SMA expression was measured using an immunocytochemistry assay, a technique used to visualize the presence of specific proteins within cells. RASMCs (5 × 10^4^ per well) were cultured into the 8-well slide chamber overnight and then pretreated with OA and DA at 250 and 500 μM for 24 h. Subsequently, they were induced with tunicamycin (1 μg/mL) in the presence or absence of OA and DA for another 24 h under serum-free medium. After 24 h of incubation, the cells were fixed with 4% PFA for 15 min, permeabilized with 0.2% Triton X-100 for 10 min, and then blocked with 3% bovine serum albumin for one hour. The slides were then incubated with primary *α*-SMA antibody (sc-58669, Santa Cruz Biotechnology, Texas, USA) at a dilution of 1:200 at 4°C overnight. Subsequently, the cells were further incubated with fluorescent secondary antibodies Alexa Fluor 488 (LGC Seracare, USA) at a dilution of 1:500 at room temperature for 1.5 h. The nucleus was stained with DAPI. Finally, images were captured using a Nikon ECLIPSE E200 fluorescence microscope (Nikon, Tokyo, Japan).

### 2.7. Quantitative Real-Time Reverse Transcription Polymerase Chain Reaction (RT-qPCR)

In this study, the gene expression of ER stress markers, including *CHOP*, *GRP78*, *ATF4*, and *eIF2α*, as well as contractile markers such as *αSMA* and *MYH11*, was assessed using real-time RT-qPCR. This technique is employed to quantify gene expression by measuring the amount of a specific RNA transcript. RASMCs (10^6^ per plates) were cultured in P100 dishes overnight and then pretreated with OA and DA at 250 and 500 μM for 24 h. After that, the cells were induced with tunicamycin (1 μg/mL) in the presence or absence of OA and DA at the same concentrations for another 24 h under serum-free medium. For RNA extraction, Sepasol-RNA I Super G (Nacalai Tesque, Inc., Kyoto, Japan) was employed, and cDNA was performed using the PrimeScript™ II 1^st^ strand cDNA Synthesis Kit (Takara Bio. Inc., Japan). Real-time PCR was performed using the Luna Universal qPCR Master (New England Biolabs Inc., Japan). The gene-specific primers used are shown in Table 1. The amplification conditions were set to 15 s at 95°C and 30 s at 62°C for 45 cycles using a real-time PCR detection system (Bio-Rad Laboratories, Inc., California, USA). GAPDH was used as the internal control, and the mRNA expression was quantified using the 2^−ΔΔCt^ method.

### 2.8. Docking

The crystal structures of human eIF2*α* and human GRP78 ATP domain were retrieved from the RCSB PDB database as PDB format files with the ascension numbers 8DYS [18] and 5EVZ [19], respectively. The water and solvent molecules as well as the native ligand, such as ATP, were then removed using AutoDockTool (ADT) [20]. Magnesium ion was retained in the GRP78. The ligand-free protein proteins were modeled using the default Gasteiger Charge in the ADT and saved in the PDBQT format. The ligands OA and DA were obtained from the PubChem database, CIDs 379 and 3893.

The ligands' energy was then minimized using the steepest descent method with a convergence of 10–12 kcal·mol^−1^ implemented in the OpenBabel software [21]. The optimized structure was converted to PDBQT format using ADT. The docking study utilized the *x*-*y*-*z* grid point numbers 90-90-90 with the macromolecule at the center. Due to our use of the binding docking assumption, these grid point numbers encompassed the protein receptor, ensuring that randomization was conducted without bias [22, 23]. The grid spacing was established at 0.375 Å. With the default parameters, a genetic algorithm (GA) with 50 iterations and a population size of 200 was carried out.

AutoDock4's Lamarckian genetic algorithm 4.2 was used to predict bound conformations [20]. As the optimal pose, the conformation with the lowest docking score in kcal·mol^−1^ was selected. Using Discovery Studio Software 2021 (BIOVIA) and the PDB structure of the ligand-protein complex, the 2D interaction between the ligand and the protein was then analyzed.

### 2.9. Statistical Analysis

All values are presented as mean values with standard error of the mean (SEM). One-way analysis of variance (ANOVA) with the Tukey's *post hoc* test was employed to determine statistical differences between the groups when the *p* value was less than 0.05.

## 3. Results

### 3.1. Cytotoxicity of OA and DA on RASMCs and RAW 264.7 Cells

**Figure 1:**
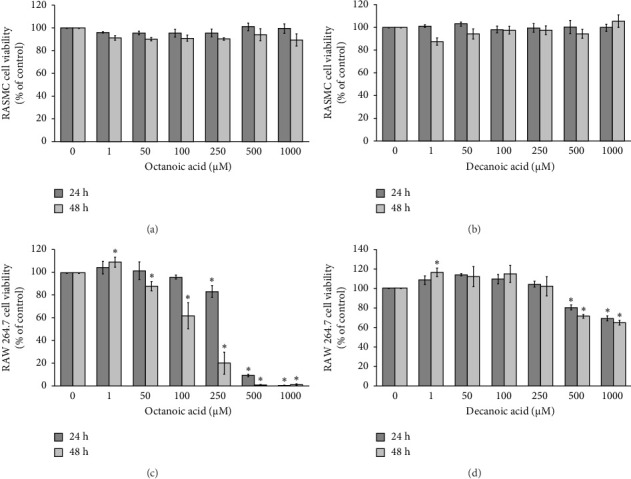
Effects of OA and DA on cell viability in RASMCs and RAW 264.7 cells. Both cells were exposed with OA and DA at the concentration of 1–1000 *μ*M for 24 and 48 h. (a, b) Cytotoxicity in RASMCs. (c, d) Cytotoxicity in RAW 264.7 cells. The data are presented as mean ± SEM from four independent experiments (*n* = 4). Statistical significance was evaluated using Tukey's *post hoc* analysis, with a significance level set at *p* < 0.05. Asterisks (⁣^∗^) indicate significant differences compared to the control group.

The cytotoxicity of RASMCs following treatment with OA and DA at concentrations of 1–1000 μM for incubation periods of 24 and 48 h is illustrated in Figure 1. The results demonstrated that none of the concentrations of OA and DA induced cytotoxicity in RASMCs at either time point when compared to the control group (Figures 1(a) and 1(b)). In contrast, cell viability significantly decreased after 24 and 48 h of OA treatment at concentrations of 100–1000 μM in RAW 264.7 cells (Figure 1(c)). DA at concentrations of 1–250 μM did not affect cell viability in RAW264.7 cells; however, concentrations of 500 and 1000 μM of DA significantly reduced cell viability after 24 and 48 h of treatment (Figure 1(d)). Consequently, we focused exclusively on RASMCs for further experiments, as both OA and DA exhibited cytotoxic effects on RAW 264.7 cells.

### 3.2. Cytoprotective Effects of OA and DA Against Tunicamycin-Induced RASMCs

**Figure 2:**
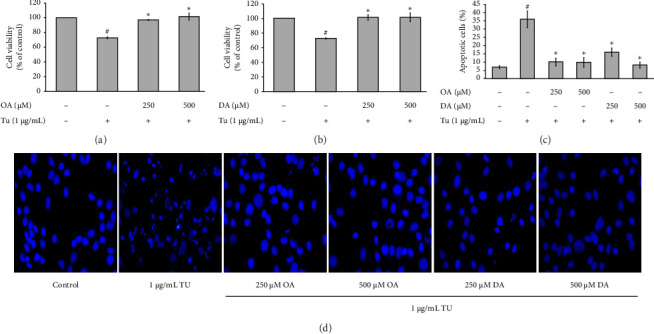
Protective effect of OA and DA against ER stress in RASMCs. (a, b) The protective effect of OA and DA against tunicamycin (TU)-induced RASMCs was evaluated using the MTT assay. (c) The percentages of apoptotic cells were then quantified and analyzed to assess the extent of apoptosis in each treatment group. (d) The morphology of apoptotic nuclei stained with DAPI was visualized using fluorescence microscopy in cells treated with OA and DA, as well as TU. All values are presented as mean ± SEM from four independent experiments (*n* = 4). Statistical significance was evaluated using Tukey's post hoc analysis, with a significance level set at *p* < 0.05. Hash symbols (^#^) indicate significant differences compared to the control group, and asterisks (⁣^∗^) indicate significant differences compared to the TU group.

To determine the protective effect of OA and DA against tunicamycin-induced ER stress in RASMCs, OA and DA were treated at concentrations of 250 and 500 μM before being exposed to tunicamycin at a concentration of 1 μg/mL. Our results demonstrated that cells pretreated with OA and DA had significantly protected them against toxicity caused by tunicamycin. The cell viability after OA treatment at 250 μM was 96.87% ± 0.56%, and that at 500 μM was 101.35% ± 4.71%, compared to 72.81% ± 1.33% viability of control group (Figure 2(a)). Similarly, DA pretreatment resulted in cell viabilities of 101.26% ± 3.56% and 101.45% ± 6.14% at 250 and 500 μM, respectively (Figure 2(b)).

For DAPI staining, our findings indicated that OA and DA pretreatment prevented RASMC apoptosis induced by tunicamycin. Without pretreatment, 36.04% ± 3.86% of cells underwent apoptosis. However, pretreating the cells with OA at 250 and 500 μM reduced apoptosis to 9.98% ± 2.40% and 9.60% ± 2.92%, respectively (Figures 2(c) and 2(d)). DA pretreatment at 250 and 500 μM reduced apoptosis to 15.95% ± 2.47% and 8.01% ± 1.88%, respectively (Figures 2(c) and 2(d)).

### 3.3. OA and DA Inhibit ROS Production Against Tunicamycin-Induced RASMCs

**Figure 3:**
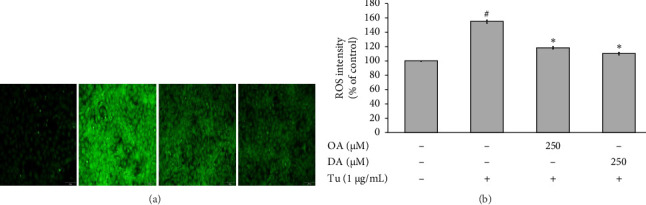
OA and DA suppress tunicamycin-induced ROS generation in RASMCs. (a) ROS levels were detected using fluorescence microscopy. (b) ROS intensity was measured using ImageJ program. All values are presented as mean ± SEM from four independent experiments (*n* = 4). Statistical significance was evaluated using Tukey's *post hoc* analysis, with a significance level set at *p* < 0.05. Hash symbols (^#^) indicate significant differences compared to the control group, and asterisks (⁣^∗^) indicate significant differences compared to the tunicamycin group.

DCFH_2_-DA probe was used to evaluate the ROS levels in RASMCs. Our finding demonstrated that OA and DA pretreated cells significantly reduced the level of ROS induced by tunicamycin, as seen in Figures 3(a) and 3(b). In contrast, tunicamycin alone significantly enhanced the ROS level compared to the cells in the control group.

### 3.4. Effects of OA and DA on ER Stress Marker Genes in Tunicamycin-Mediated Apoptosis in RASMCs

**Figure 4:**
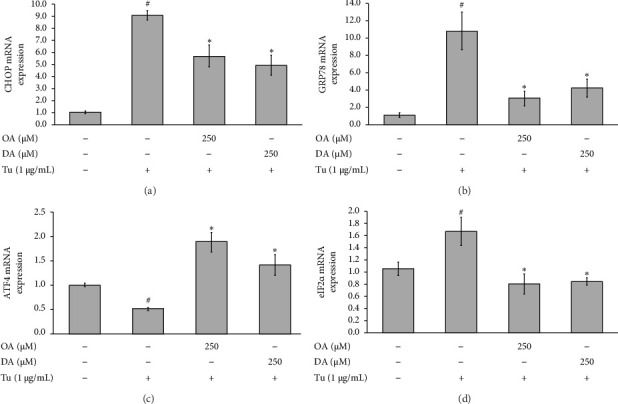
Effects of OA and DA on the mRNA expression of CHOP, GRP78, ATF4, and eIF2*α* on tunicamycin-induced RASMCs. The cells were pretreated with OA and DA for 24 h followed by tunicamycin for 24 h. (a) CHOP. (b) GRP78. (c) ATF4. (d) eIF2*α*. All values are presented as mean ± SEM from four independent experiments (*n* = 4). Statistical significance was evaluated using Tukey's *post hoc* analysis, with a significance level set at *p* < 0.05. Hash symbols (^#^) indicate significant differences compared to the control group, and asterisks (⁣^∗^) indicate significant differences compared to the tunicamycin group.

The mRNA expressions levels of CHOP, GRP78, ATF4, and eIF2*α* were determined by real-time RT-PCR, as presented in Figure 4. Tunicamycin increased the expression level of CHOP, GRP78, and eIF2*α* by approximately 9.08 ± 0.38, 10.82 ± 2.16, and 1.67 ± 0.23, respectively, while decreasing the expression of ATF4 by 0.50 ± 0.03 (Figures 4(a), 4(b), 4(c), and 4(d)). However, pretreating the cells with OA and DA significantly suppressed the gene expression of *CHOP*, *GRP78*, and *eIF2α* and increased the gene level of *ATF4* compared to the tunicamycin treatment alone (Figures 4(a), 4(b), 4(c), and 4(d)).

### 3.5. OA and DA Prevent ER Stress–Induced RASMC Injury by Decreasing Contractile Markers

**Figure 5:**
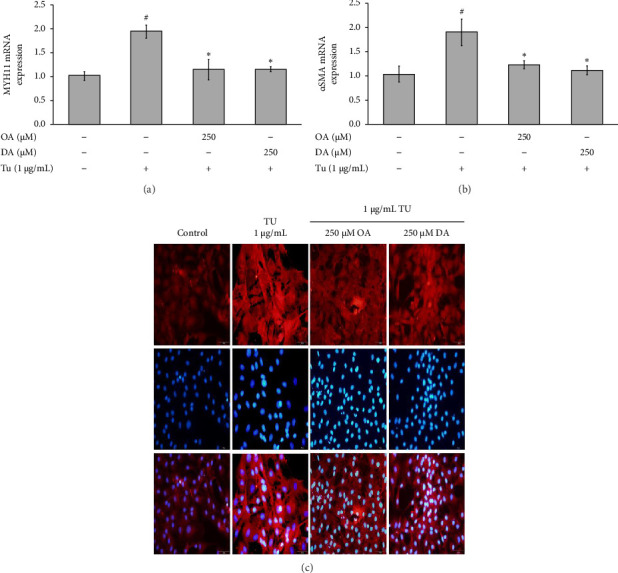
Effects of OA and DA on the expression of MYH11 and *α*SMA on tunicamycin-induced RASMCs. (a) MYH11, (b) *α*SMA, and (c) *α*SMA proteins were determined using immunofluorescence. Statistical significance was evaluated using Tukey's *post hoc* analysis, with a significance level set at *p* < 0.05. Hash symbols (^#^) indicate significant differences compared to the control group, and asterisks (⁣^∗^) indicate significant differences compared to the tunicamycin group.

The levels of *α*SMA were assessed by real-time RT-PCR and immunocytochemistry, whereas *MYH11* gene expression was detected by real-time RT-PCR, as shown in Figure 5. The results showed that tunicamycin significantly induced the expression level of *α*SMA and MYH11 by approximately 1.90 ± 0.28, and 1.93 ± 0.14, respectively, compared to the control group (Figures 5(a) and 5(b)). Both pretreatments of OA and DA significantly downregulated the expression level of *α*SMA and MYH11 in RASMCs compared with the tunicamycin treatment alone (Figures 5(a) and 5(b)). To investigate the impact of OA and DA on *α*SMA, immunocytochemistry analysis was conducted to identify *α*SMA protein in cells treated with OA and DA, either with or without tunicamycin. The results, as shown in Figure 5(c), indicate that pretreating cells with OA and DA before exposing them to tunicamycin significantly reduced the number of *α*SMA-positive cells compared to tunicamycin alone.

### 3.6. Docking

**Figure 6:**
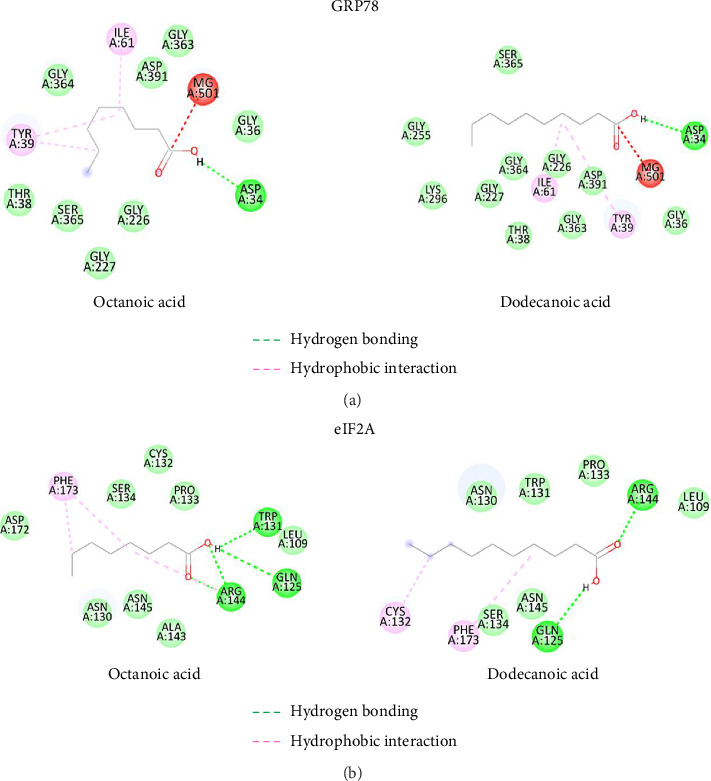
Interaction scheme of the selected fatty acids with GRP78 (a) and eIF2A (b). The green and pink dash lines denoted the hydrogen bonding and hydrophobic interactions.

Both OA and DA bound to eIF2A with comparable docking scores, but DA scored slightly higher than OA with the ATP-free GRP78 (Table 2). Docking analyses revealed that both fatty acids favored eIF2A over ATP-free GRP78 for binding. Interactions with the pocket site were caused by hydrogen bonding from the carboxylic group and hydrophobic interaction from the fatty acid chain (Figures 6(a) and 6(b)). The remarkably comparable docking score between these compounds may be due to the similar number of carbons in the sidechain and the sidechain's adaptability to the binding site.

Both fatty acids were bound to the eIF2A site via hydrogen bonding between the carboxylic group and glutamine 125 (Gln125) and arginine 144 (Arg144) as depicted in Figure 6(b). In addition to hydrophobic interactions, phenylalanine 173 (Phe173) showed hydrophilic interactions. Both fatty acids were bound to the same magnesium-containing GPR78 site (Figure 6(a)). Docking revealed hydrogen bonds between the carboxylic group and aspartic acid 34 (Asp34) as well as hydrophobic interactions with tyrosine 39 (Tyr39).

To be noted, the docking was performed to see if it is possible for the OA and DA could bind eIF2*α* and GRP78. If it can bind, the effect of the downstream molecules will be changed, but the binding could not affect the expression level of these two proteins.

## 4. Discussion

Oxidative stress plays a pivotal role in numerous vascular diseases, including aortic aneurysm, atherosclerosis, and ischemia-reperfusion injury [24, 25]. Moreover, ROS can induce injury and apoptosis in VSMCs [26, 27]. Additionally, the relationship between ER stress and cellular oxidative status was also explored. The induction of ER stress was found to trigger ROS production, which in turn contributed to ROS-dependent ER stress and ultimately led to the death of VSMCs [27, 28].

The ER, in collaboration with the nuclear envelope, plays a crucial role in protein maturation, calcium regulation, and lipid synthesis. During post-translational modification, the primary role of the ER is to regulate the proper folding of proteins within its lumen [8]. Additionally, ER stress–related impairments in cellular function and ER stress–induced programmed cell death also contribute to the development and progression of various human diseases [8, 9]. Vascular cells are particularly susceptible to ER stress–induced damage, which can lead to shear stress within blood vessel lumens, ultimately promoting inflammation in VSMCs [28, 29]. Shear stress has also been shown to induce apoptosis in human VSMCs through an ER stress–dependent mechanism [29]. Various factors, including high blood glucose, trigger ER stress in VSMCs, while pathological conditions, inflammation, and ROS disruption are associated with increased ER stress and impaired VSMC function [30]. By reducing ROS production, these cells may be protected from ER stress–induced damage, offering a potential preventive strategy against cardiovascular diseases [31]. Our study is the first evidence of the novel protective role of OA and DA in mitigating ER stress–stimulated cell injury in RASMCs by reducing ROS generation. Pretreating cells with OA and DA before inducing ER stress effectively maintained cell survival and suppressed cell injury. Furthermore, this finding indicates that alleviating ER stress–induced apoptosis is closely associated with a decrease in ROS generation in RASMCs. Additionally, our study revealed that the application of nontoxic concentrations of OA and DA as pretreatment reduced CHOP levels compared to the tunicamycin group. The observation that OA and DA inhibit ER stress–mediated apoptosis suggests that these compounds modulate CHOP-dependent signaling pathways, thereby reducing the likelihood of cell death. By targeting these pathways, OA and DA may help alleviate the detrimental effects of ER stress and promote cellular survival. The activation of eIF2*α* triggers the activation of ATF4, which subsequently enhances the production of proteins involved in protein folding and degradation processes [8]. Emerging research indicates that the activation of the protein kinase RNA-like endoplasmic reticulum kinase pathway during ER stress can induce apoptosis through a mechanism involving the transcription factor ATF4 [32]. The ER chaperone GRP78 plays a pivotal role as a regulator of ER stress. It contributes significantly by promoting efficient ER activity in the assembly of nascent polypeptides, thus preventing protein misfolding and aggregation [33]. Furthermore, GRP78, a molecular chaperone, recognizes and binds to misfolded proteins, preventing their aggregation and facilitating their degradation by the proteasome, which contributes to restoring ER homeostasis [34]. Our research shows that pretreating RASMCs with OA and DA significantly reduced the elevation of eIF2*α* and GRP78 levels in response to tunicamycin. Furthermore, the pretreatment with OA and DA substantially elevated ATF4 levels compared to the tunicamycin group. This finding suggests that OA and DA play a role in mitigating ER stress–mediated apoptosis by reducing the activation of the ATF4, eIF2*α*, and GRP78 pathways.

In patients with abdominal aortic aneurysm, VSMCs have been found to exhibit impaired contraction compared to nonpathologic control VSMCs [5]. In response to mechanical stress, VSMCs exhibit increased expression of contractile proteins, including *α*-SMA and MYH11. These cells are essential for maintaining the structural integrity of the aortic wall [6]. In the current study, the expression of *α*-SMA and MYH11 significantly increased in response to tunicamycin, an ER stress inducer. However, pretreatment with OA and DA led to a decrease in the levels of both *α*-SMA and MYH11 expressions in RASMCs. In this context, the current study enhances the understanding of abdominal aortic aneurysm pathology related to ER stress in RASMCs. It demonstrates that ER stress, characterized by increased levels of CHOP, GRP78, and eIF2*α*, plays a role in regulating ROS production and inducing apoptosis. This process, in turn, contributes to the elevation of contractile proteins (*α*-SMA and MYH11) and the occurrence of vascular injury. Nevertheless, it is essential to note that further investigations are required. We hypothesize that OA and DA may serve as beneficial nutrients in safeguarding VSMCs in abdominal aortic aneurysm. The regulation of various factors in VSMCs within the aorta is crucial in preventing the development of abdominal aortic aneurysm and influencing the prognosis of this condition.

## 5. Conclusion

The current investigation has unveiled how OA and DA possess the capacity to safeguard RASMCs from ER stress–induced apoptosis. This protective effect is achieved through the reduction of ROS production, the inhibition of CHOP, GRP78, and eIF2*α*, and the enhancement of ATF4 levels, all of which collectively contribute to the preservation of contractile proteins, including *α*-SMA and MYH11. These findings have significant implications for the development of innovative therapeutic approaches and novel compounds targeting VSMC-related disorders.

## Figures and Tables

**Table 1:** Primer sequence.

Gene	Forward primer	Reverse primer
GAPDH	5′-CTCTCTGCTCCTCCCTGTTC-3′	5′-TACGGCCAAATCCGTTCACA-3′
CHOP	5′-GAGGAGAGAGAAACCGGTCCAAT-3	5′-GGTGCCCCCAATTTCATCTG-3′
GRP78	5′-CGCTCGATACTGGCTGTGAC-3′	5′-CCGTGCCTACATCCTCCTTC-3′
ATF4	5′-CGGAACGTGACTTCAGTTGG-3′	5′-TATCCCATTGACGCTCTGGC-3′
eIF2A	5′-TGCTACACCTTTGTGAGTGTG-3′	5′-TCCATTGCTCCAGGCAAACA-3′
MYH11	5′-CTCCCAACCATCAACTGCCT-3′	5′-TTCGTGAAAGTGTCCGTGGG-3′
*α*SMA	5′-GGATCAGCGCCTTCAGTTCT-3′	5′-GGGCTAGAAGGGTAGCACAT-3′

**Table 2:** Docking score of the ligand toward eIF2A and GRP78 via molecular docking.

Ligand	Docking score in kcal·mol^−1^	
	eIF2A	GRP78
Octanoic acid	−4.98	−9.17
Decanoic acid	−4.88	−9.65

## Data Availability

The data are available from the authors on request.

## Nomenclature

ATF4: Activating transcription factor 4
CHOP: CCAAT-enhancer-binding protein homologous protein
DA: Decanoic acid
ER: Endoplasmic reticulum
eIF2*α*: Eukaryotic initiation factor 2 alpha
GRP78: Glucose regulatory protein 78
MCFAs: Medium-chain fatty acids
MYH11: Myosin heavy chain 11
OA: Octanoic acid
ROS: Reactive oxygen species
*α*-SMA: *α*-Smooth muscle actin
VSMCs: Vascular smooth muscle cells
